# Deep learning enhancing guide RNA design for CRISPR/Cas12a‐based diagnostics

**DOI:** 10.1002/imt2.214

**Published:** 2024-06-15

**Authors:** Baicheng Huang, Ling Guo, Hang Yin, Yue Wu, Zihan Zeng, Sujie Xu, Yufeng Lou, Zhimin Ai, Weiqiang Zhang, Xingchi Kan, Qian Yu, Shimin Du, Chao Li, Lina Wu, Xingxu Huang, Shengqi Wang, Xinjie Wang

**Affiliations:** ^1^ Zhejiang Lab Hangzhou China; ^2^ Department of Laboratory Medicine, The First Affiliated Hospital Zhejiang University School of Medicine Hangzhou China; ^3^ Key Laboratory of Clinical In Vitro Diagnostic Techniques of Zhejiang Province Hangzhou China; ^4^ Institute of Laboratory Medicine Zhejiang University Hangzhou China; ^5^ Department of Applied Mathematics and Theoretical Physics University of Cambridge Cambridge UK; ^6^ School of Medicine, School of Science and Engineering University of Dundee, Nethergate Dundee UK; ^7^ School of Food Science and Pharmaceutical Engineering Nanjing Normal University Nanjing China; ^8^ Bioinformatics Center of AMMS Beijing China; ^9^ Shenzhen Branch, Guangdong Laboratory of Lingnan Modern Agriculture, Genome Analysis Laboratory of the Ministry of Agriculture and Rural Affairs, Agricultural Genomics Institute at Shenzhen Chinese Academy of Agricultural Sciences Shenzhen China

**Keywords:** Cas12a, CRISPR, crRNA design, convolutional neural network, deep learning, diagnostic

## Abstract

Rapid and accurate diagnostic tests are fundamental for improving patient outcomes and combating infectious diseases. The Clustered Regularly Interspaced Short Palindromic Repeats (CRISPR) Cas12a‐based detection system has emerged as a promising solution for on‐site nucleic acid testing. Nonetheless, the effective design of CRISPR RNA (crRNA) for Cas12a‐based detection remains challenging and time‐consuming. In this study, we propose an enhanced crRNA design system with deep learning for Cas12a‐mediated diagnostics, referred to as EasyDesign. This system employs an optimized convolutional neural network (CNN) prediction model, trained on a comprehensive data set comprising 11,496 experimentally validated Cas12a‐based detection cases, encompassing a wide spectrum of prevalent pathogens, achieving Spearman's *ρ* = 0.812. We further assessed the model performance in crRNA design for four pathogens not included in the training data: Monkeypox Virus, Enterovirus 71, Coxsackievirus A16, and *Listeria monocytogenes*. The results demonstrated superior prediction performance compared to the traditional experiment screening. Furthermore, we have developed an interactive web server (https://crispr.zhejianglab.com/) that integrates EasyDesign with recombinase polymerase amplification (RPA) primer design, enhancing user accessibility. Through this web‐based platform, we successfully designed optimal Cas12a crRNAs for six human papillomavirus (HPV) subtypes. Remarkably, all the top five predicted crRNAs for each HPV subtype exhibited robust fluorescent signals in CRISPR assays, thereby suggesting that the platform could effectively facilitate clinical sample testing. In conclusion, EasyDesign offers a rapid and reliable solution for crRNA design in Cas12a‐based detection, which could serve as a valuable tool for clinical diagnostics and research applications.

## Highlights

- Advanced crRNA design system: Developed EasyDesign, utilizing a convolutional neural network (CNN) trained with over 11,000 diagnostic‐target pairs to enable the creation of highly sensitive crRNAs for Cas12a‐based nucleic acid diagnostics.
- Proven predictive capabilities: EasyDesign demonstrates superior predictive performance for crRNA‐mediated Cas12a detection, validated through its successful application in designing diagnostics for a variety of viruses in clinical settings.
- User‐friendly web platform: Features an intuitive web platform that streamlines the creation of CRISPR/Cas12a‐based diagnostic tools, thereby enhancing accessibility and usability for both researchers and clinicians.

## Graphical abstract

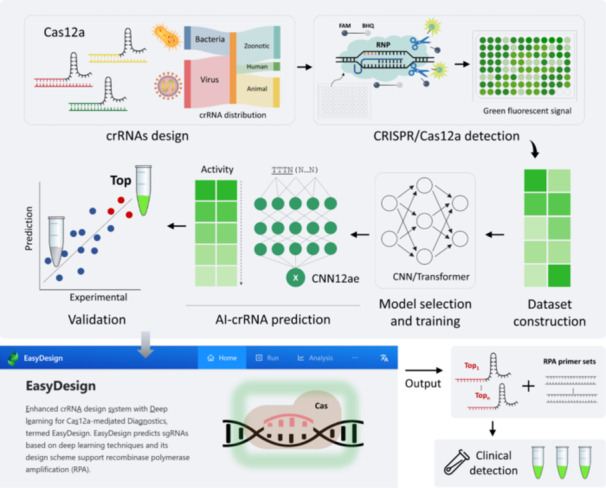

## INTRODUCTION

Rapid and accurate diagnostic tests for infectious diseases play an essential role in safeguarding public health and providing timely care to individual patients [1]. Recent outbreaks such as COVID‐19 and monkeypox have highlighted the urgent need for effective diagnostic tools [2, 3]. The Clustered Regularly Interspaced Short Palindromic Repeats (CRISPR) and CRISPR Associated Protein (Cas)‐based detection system, using Cas12 or Cas13 enzymes, serves to identify specific nucleic acid sequences, subsequently activate nonspecific collateral cleavage capabilities for downstream readout processes [4, 5]. With the advantages of rapidity, specificity, sensitivity, and ease of use, CRISPR/Cas‐based detection has emerged as a promising approach for point‐of‐care testing (POCT) of infectious diseases [6].

The design of effective CRISPR RNA (crRNA) is critical for the CRISPR/Cas‐based diagnostic system. It improves the pairing of crRNAs and their target sequences, reducing off‐target effects and improving overall efficiency [7]. However, the current design requires the determination of a protospacer adjacent motif (PAM) sequence, and specificity and sensitivity testing, which is time‐consuming, expensive, and may be biased by personal experience. Therefore, there is an urgent need for a rapid and efficient method to design crRNAs with high specificity and sensitivity [8, 9]. The development of computational approaches promises to streamline the process, saving time and resources while improving accuracy.

Deep learning has emerged as a powerful tool for designing crRNAs for CRISPR‐based genome editing systems [10]. Convolutional neural networks (CNNs) and recurrent neural networks (RNNs), trained on large datasets of experimentally validated crRNAs, have shown superior performance in predicting crRNA activity and specificity [11, 12]. In addition, recent studies have shown that deep neural networks outperformed other models in designing Cas13 diagnostic systems [9] and Cas12 editing systems. Notably, deep learning approaches have successfully predicted the activities of guide RNA, such as AsCpf1 [13] and Cpf1 [14]. However, a validated model for Cas12a‐based diagnostics is still lacking, which promises to accelerate the development of Cas12a‐based rapid diagnostics for pathogens. Furthermore, challenges remain in translating deep learning models into real‐world diagnostic practice, facilitating its widespread adoption in clinical and research applications, as all existing models are developed based on synthetic libraries that do not reflect the genome of real‐world pathogens.

In this study, we present EasyDesign (enhanced crRNA design system with deep learning for Cas12a‐mediated diagnostics), a CNN‐based crRNA design system tailored for CRISPR/Cas12a‐based diagnostics. EasyDesign employs a CNN‐based model trained on a data set derived from high‐quality Cas12a detection sequences covering common bacterial and viral pathogens. We demonstrate that the crRNAs produced by EasyDesign exhibit exceptional efficiency in the detection of Monkeypox Virus (MPXV), Enterovirus 71 (EV71), Coxsackievirus A16 (CV‐A16), *Listeria monocytogenes* (*L. monocytogenes*), and human papillomavirus (HPV). In addition, EasyDesign provides a user‐friendly web server (https://crispr.zhejianglab.com/) that integrates recombinase polymerase amplification (RPA) primer and crRNA design.

## RESULTS

### Data characteristics for modeling and model evaluation methods

**Figure 1:**
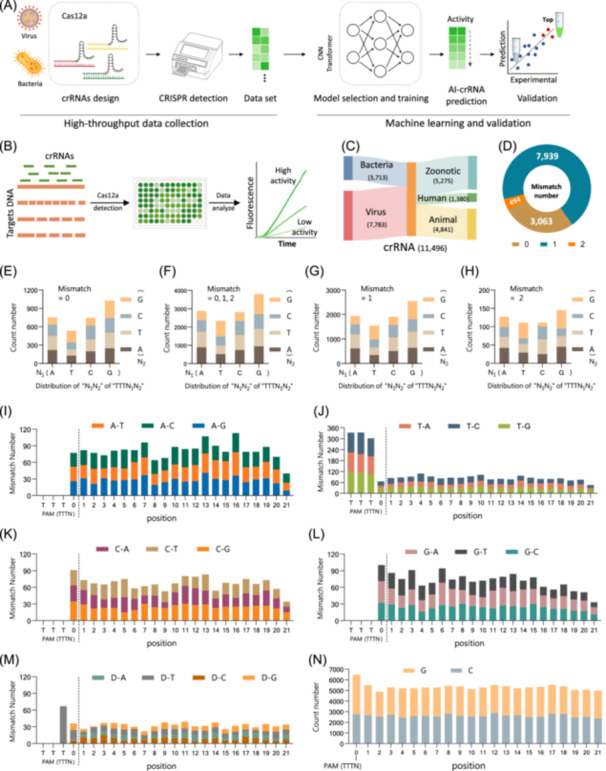
Preparation and evaluation of the Cas12a‐based crRNA data set for deep learning models training. (A) The process of developing deep learning‐based crRNA design models, including high‐throughput data collection, machine learning, and validation. (B) Flowchart illustrating the process of data acquisition using the CRISPR fluorescence‐based assay. (C) Distribution of the number of mismatches in the data set for guide‐to‐target pairs, including 0, 1, and 2 mismatches. (D) Characterization of the distribution of pathogens in the training data set, including viruses and bacteria. (E)–(H), Distribution of base types denoted as “N_1_N_2_” within the “TTTN_1_‐N_2_” region of the protospacer adjacent motif (PAM) and its adjacent extending position. (I)–(M) Types of mutations in the guide‐to‐target pairs, including A‐N, T‐N, C‐N, G‐N, and deletion (D)‐N. (N) Distribution of the GC content at different positions within the crRNA data set. crRNA, CRISPR RNA; CRISPR, Clustered Regularly Interspaced Short Palindromic Repeats.

Our study includes stages of data acquisition, model training, and validation (Figure 1A). (1) To develop the training datasets for the deep learning models, we prepared high‐quality sequences of Cas12a crRNA along with the corresponding fluorescence detection. We designed crRNAs targeting a variety of pathogenic nucleic acid sequences, covering the primary pathogens outlined by the World Health Organization (WHO) [15] and World Organization for Animal Health (WOAH) [16], particularly considering the public health threat posed by pathogens of animal origin [17, 18, 19]. We then applied a previously established fluorescent CRISPR methodology using a microplate reader for fluorescence detection [20]. The resulting fluorescence readouts, together with the DNA and crRNA sequences, formed the training data set. (2) Deep learning models were trained and evaluated, including CNN, Transformer, and enhanced version. The model exhibiting the highest Spearman's rank correlation coefficient (*ρ*) in predicting crRNA activity was carefully selected for subsequent model validation on unseen nucleic acid data. The consistency between the model predictions and the ground truth experimental outcomes is a testament to the robustness and reliability.

### High‐quality datasets of crRNA mediate Cas12a detection

To establish a training data set representative of real‐world diagnostic practice, we designed 1533 crRNAs targeting 34 natural bacterial and viral pathogens (Table S1), covering the common pathogens identified by the WHO and WOAH. We introduced random mutations into the original pathogen templates to account for the frequent evolutionary mutations inherent in these pathogens, resulting in 198 DNA templates (Table S2).

Subsequently, we employed a crRNA‐mediated Cas12a detection as previously described [20], collecting fluorescence readouts at 2‐min intervals throughout a 2‐h experiment to assess the reactivity of the crRNA library (Figure 1B). We selected 11,496 fluorescence readouts obtained at the 30‐min mark as the training data set. The generated guide‐to‐target pairs include 7783 pairs from viral sources and 3713 pairs from bacterial sources. Of these, 5275 pairs are zoonotic pathogens, 1380 pairs are human pathogens, and 4841 pairs are animal pathogens (Figure 1C).

To ensure the quality of the generated guide‐to‐target pairs, we further analyzed the mismatches in these pairs and identified 3063 pairs with no mismatches, 7939 pairs with one‐base mismatches, and 494 pairs with two‐base mismatches, respectively. The average mismatch among all guide‐to‐target pairs was 0.766, indicating a reasonable mixture of wild‐type and mutant pairs, reflecting the natural mutation probability [21] (Figure 1D). Subsequently, we examined the distribution of bases in all guide‐to‐target pairs and observed a uniform distribution of base types (Figure 1E–H). Among all guide‐to‐target pairs containing mismatches, base mutations included A‐N, T‐N, C‐N, G‐N, and deletion‐N, with no significant bias observed in these mutation types (Figure 1I–M). For all the crRNAs, the distribution of base types at the 21 positions downstream of the PAM remained relatively consistent (Figures 1N and S1).

In the data set, we verified that a high degree of mismatch leads to reduced activity (Figure S2). This finding supports the notion that generating high‐mismatch data improves model training. Mechanistically, the presence of poly(T) at the 5′ end of the crRNA appeared to be associated with a decrease in reactivity. As expected, the data indicated a substantial decrease in activity when the “N” in the PAM (TTTN) was “T,” further demonstrating that “TT” exhibited lower activity compared to TA, TC, and TG (Figure S3A). Furthermore, we investigated the effect of mismatch on the activity of the guide‐to‐target pairs. As expected, the results indicated that guide‐to‐target pairs without mismatches exhibited significantly higher activity than those with mismatches (Figure S3B). In addition, no significant effect on activity was observed from the mutation of “N” in the PAM sequence (TTTN), except for the notable effect of base deletions at specific positions (Figure S3C). These features further support the generalizability of our training data. Following these quality checks, the guide‐to‐target pairs and corresponding fluorescence data constitute the datasets for training the deep learning models, including the training data (Table S3), the augment data (Table S4), and the test data (Table S5).

### Development of a deep learning model for efficient crRNA design

**Figure 2:**
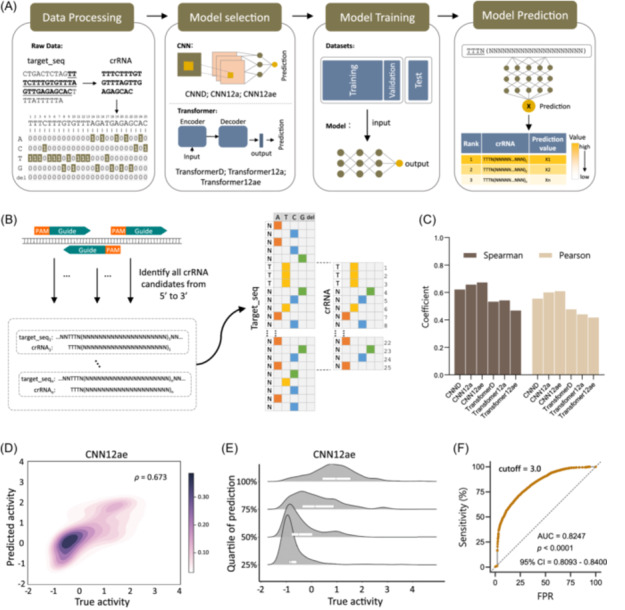
Development and evaluation of a deep learning model suitable for Cas12a diagnostic design. (A) A four‐step flowchart outlining the process of selecting and training deep learning models. (B) The performance comparison of models using the Spearman's correlation coefficient and Pearson's correlation coefficient. (C) The CNN12a model uses one‐hot encoding for all enumerated crRNA‐target DNA sequence pairs with a “TTTN” PAM. (D) The Kernel density estimation of true (*x*‐axis) and predicted values (*y*‐axis) of CNN12ae. (E) The density map of true activity by quartile of predicted value; *x*‐axis is the normalized true activity; *y*‐axis is the interquartile range of predictions; it shows that the distribution of true activity is more concentrated when the CNN12a predicted value is below the second quartile. (F) Receiver operating characteristic (ROC) curve for the hold‐out test set of CNN12ae, distinguishing pairs as either inactive or active (true activity = 3), with an AUC (area under the curve) of 0.8247 (*p* < 0.0001). CNN, convolutional neural network; crRNA, CRISPR RNA; FPR, false positive rate; PAM, protospacer adjacent motif.

To train the deep learning models, we designed a four‐step process including data processing, model selection, training, and validation (Figure 2A). First, we used a one‐hot (2D) encoding for the base encoding. Then, we tested two reported good‐performing models, CNN‐derived [9] (CNND) and Transformer‐derived [22] (TransformerD) models. We further trained two models, namely CNN12a and Transformer12a, by adapting CNND and TransformerD models to the Cas12a diagnostic scenarios (Figure S4). Finally, the optimal model was used to predict the activity of crRNAs.

During the crRNA candidate design process, we conducted an iterative search for the PAM target sequence (TTTN) and subsequently extracted a 21‐nucleotide sequence downstream of the PAM site. To account for potential influences from the surrounding sequence, we extended the crRNA by an additional 10 nucleotides at both the 5′ and 3′ terminals of the target, resulting in a 45‐nucleotide target sequence (Figure 2B). The data set of 11,496 fluorescence readouts was divided into a training set of 10,634 pairs and a separate test set of 862 pairs, with the stipulation that the test set excluded the target sequences used in the training set. The CNN and Transformer models were trained on the Cas12a detection data set, based on previous crRNA efficiency prediction [12]. All four trained models were evaluated using the test set. Notably, the CNN12a and Transformer12a models, which were tailored to Cas12a features and yielded higher Spearman's rank correlation coefficients (*ρ* values: 0.656 and 0.541, respectively), showed improved performance over the CNND and TransformerD models (*ρ* values: 0.620 and 0.532, respectively, Figure 2C).

We implemented data augmentation in the training process to further improve model performance. Specifically, we used and normalized 20‐min and 30‐min readouts from the same assay to create an augmented data set, containing 31,993 guide‐to‐target pairs (see Methods section for details). The CNN12a and Transformer12a models were then trained using this augmented data set, leading to CNN12ae and Transformer12ae models, respectively. Upon evaluation using the test set, we found that CNN12ae achieved improved performance over the CNN12a model (Spearman's *ρ* = 0.673) and also outperformed Transformer12ae (*ρ* = 0.467) (Figure 2C). Notably, CNN12ae achieved improved performance (Spearman's *ρ* = 0.812) on a larger test set that included both the original test data and an additional 30% of guide‐to‐target pairs with high mismatches.

We performed kernel density estimation and actual activity analysis on the predicted values to evaluate the performance of CNN12ae and other models in the hold‐out test. We found that CNN12ae showed distinct regions characterized by different levels of activity compared to other models (Figures 2D and S5). Notably, CNN12ae demonstrated superior performance in the low‐value region (Figures 2E and S5). Additionally, we assessed the classification performance of the CNN12ae model, which yielded an AUC value of 0.8247 with a *p* value < 0.0001, indicating strong classification capabilities (Figure 2F). We found that the predictive performance of CNN12ae was generally consistent for viral (0.66) and bacterial (0.69) pairs (Figure S6), supporting the generalization properties of the model. Furthermore, the CNN12ae was evaluated using a five‐fold cross‐validation approach on a data set of 33,351 data points, ensuring strict segregation of target RNA sequences between training and testing sets to prevent data leakage. Each of the five‐folds, containing 26,952 training and 6399 testing data points, underwent 450 epochs of training. Model efficacy was gauged by Spearman's and Pearson's correlation coefficients, yielding Spearman's values of 0.8038, 0.8267, 0.7962, 0.7939, and 0.8481, and Pearson's values of 0.7103, 0.7399, 0.7202, 0.6649, and 0.7878 across the folds. These consistent and high correlation coefficients suggest that the model possesses robust generalizability, indicating its potential for accurate predictions in its respective field (Table S6). Consequently, we selected the CNN12ae model for further Cas12a‐mediated crRNA design.

### Evaluation of EasyDesign on pathogenic nucleic acid detection

**Figure 3:**
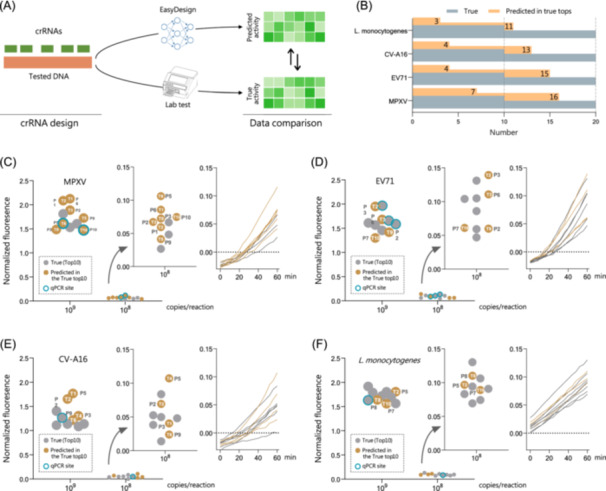
Validation of EasyDesign performance across four pathogens. (A) Flowchart illustrating the experimental validation of EasyDesign through a comparative screening test on pathogen templates. (B) Comparison between the experimentally detected number of top crRNAs and the number of top crRNAs predicted by EasyDesign. (C)–(F) Comparative analysis of crRNA activity in experimental CRISPR fluorescence versus EasyDesign predictions, including Monkeypox Virus (MPXV), Enterovirus 71 (EV71), Coxsackievirus A16 (CV‐A16), and *Listeria monocytogenes* (*L. monocytogenes*). CRISPR, Clustered Regularly Interspaced Short Palindromic Repeats; crRNA, CRISPR RNA.

To validate the applicability of the developed model, we performed experimental crRNA activity, and EasyDesign predicted crRNA ranking for four pathogenic nucleic acid sequences (Figure 3A and Table S7), 194 crRNA in total (Table S8). These sequences include MPXV, a pathogen responsible for a recent outbreak; EV71 and CV‐A16, which cause severe hand, foot, and mouth disease in children; *L. monocytogenes*, a foodborne bacterial pathogen known to cause severe disease in humans.

Among the crRNA candidate sets for the four pathogens, we found consistency between the crRNAs predicted by the deep learning model and the experimentally verified ground‐truth crRNAs, demonstrated in 7, 4, 4, and 3 cases from each pathogen for the top 10 crRNAs (Figure 3B); whereas 16, 15, 13, and 11 cases for the top 20 crRNAs. These results suggest that the model could efficiently identify highly reactive crRNAs for experimental assays from a limited number of predicted candidates, significantly reducing the screening intensity.

Furthermore, our experimental results showed that the predicted crRNAs generated by the deep learning model exhibited equal or higher reactivity than the reported quantitative polymerase chain reaction (qPCR) sites, particularly at a target DNA concentration of 10^9^ copies/reaction (Figures 3C–F and S7). Notably, when using lower DNA template concentrations (10^8^ copies/reaction), variations in crRNA activity rankings were generally consistent with expectations, albeit slightly variable. These findings indicate that EasyDesign could predict crRNAs with high activity, providing valuable insights for efficient crRNA selection.

### User‐friendly web tool for End‐to‐End Cas12a diagnostic design

**Figure 4:**
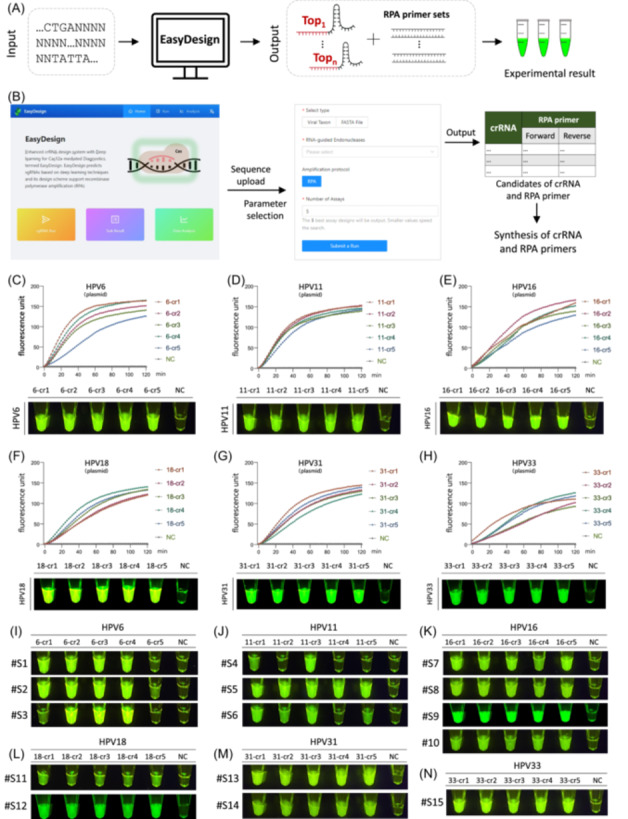
HPV clinical sample testing design via EasyDesign web server. (A) Flowchart illustrating the web‐based EasyDesign platform, including sequence input, crRNA, and amplification primer design for Cas12a‐based detection. (B) Presentation of the web‐based design interface, including sequence uploading, parameter selection, and the generation of candidate crRNA and amplification primer pairs. (C)–(H) Fluorescence detection kinetic curves representing six human papillomavirus (HPV) subtypes (HPV6, HPV11, HPV16, HPV18, HPV31, and HPV33) using synthetic DNA templates. The optimal amplification primers and crRNAs were generated utilizing the EasyDesign web‐based tool. Five optimal crRNAs and their corresponding primer pairs were generated for each template. The fluorescence graph illustrates the results obtained after a 30‐min incubation period. (I)–(N) Fluorescence detection results were obtained for clinical samples representing the six HPV subtypes after a 30‐min incubation period. The positive clinical samples were identified as follows: #S1 to #S3 for HPV6, #S4 to #S6 for HPV11, #S7 to #S10 for HPV16, #S11 and #S12 for HPV18, #S13 and #S14 for HPV31, and #S15 for HPV33. crRNA, CRISPR RNA; NC, negative control.

To improve the efficiency of Cas12a diagnostic development, we have created an online service, EasyDesign, which can be accessed at https://crispr.zhejianglab.com/. This platform provides a comprehensive Cas12a‐based diagnostic design experience that seamlessly integrates RPA primer design, facilitating the development of RPA‐CRISPR assays with recommended crRNAs and RPA primers (Figure 4A). Our web‐based platform has a user‐friendly interface that guides users through each step of the workflow, including sequence input, parameter selection, and crRNA design output. In particular, EasyDesign offers annotated design visualizations that elucidate the interaction of crRNAs and primers with the target sequence.

### EasyDesign facilitates HPV clinical sample diagnostic

To evaluate the effectiveness of EasyDesign, we used it to design an RPA‐CRISPR assay for clinical samples of six HPV subtypes, including subtypes HPV6, HPV11, HPV16, HPV18, HPV31, and HPV33. The optimal crRNAs and recommended RPA primers were synthesized (Table S9) for each of the six HPV subtypes as generated by EasyDesign (Figure 4B). Using the input template DNA sequence and the combination of candidate crRNAs and RPA primers provided by our online server, we achieved a robust fluorescent detection signal for synthetic DNA templates of HPV (Figure 4C–H). When detecting different subtypes of clinical samples, we observed a significant fluorescence signal for all five candidate crRNAs (Figure 4I–N and S8), indicating the effectiveness of EasyDesign. Although EasyDesign enables the accurate prediction of highly active crRNAs, it could facilitate the development of Cas12a‐based detection for the research community worldwide via the online service.

## DISCUSSION

CRISPR/Cas12a‐based diagnostic assays provide a platform that combines the advantages of qPCR and serological assays in terms of portability, specificity, and ease of use [23]. More recently, these platforms have been rapidly shifted to aid in detecting SARS‐CoV‐2, with assays using both enzymes gaining FDA approval [24]. The broader implementation of assays using both CRISPR/Cas12a has been significantly limited by the tedious design process required for primers and crRNA guides. The design process challenges researchers to ensure that their chosen targets have sequence conservation in both the chosen primer and crRNA target sequences. Both issues lead to reduced cleavage efficiency and lower detection sensitivity. In this study, we developed EasyDesign, an internal tool to rapidly accelerate the design of CRISPR/Cas12a assay targets.

Precision‐oriented deep learning models require a foundation built on rigorously accurate detection data. To address this evolving challenge, we first embarked on a mission to capture an expansive array of reactions. We have meticulously designed the DNA templates of 35 pathogens, enabling a global design of crRNAs. This step is essential to collect robust assay data to support the development of our deep learning model. Our comprehensive data pool includes diverse viruses and bacteria, including zoonotic pathogens, human target viruses, and animal viruses. This broad coverage increases the generalizability of our data, offering a more authentic representation of nucleic acid distribution characteristics found in different biological species. We then used our previously established CRISPR fluorescence assay to execute a high throughput testing [20]. The challenge that emerges here lies in the normalization of the data. To ensure consistency and reliability, we strategically positioned the same positive and negative controls on each 96‐well plate. This measure allows us to harmonize the data across all samples, which is an invaluable resource in our quest to develop accurate deep‐learning models.

When responding to emerging outbreaks, rapid and accurate design of pathogenic nucleic acid testing protocols and easy‐to‐use assays are essential to prevent the spread of the virus and provide effective medical treatment, especially in areas where the disease is endemic or where outbreaks have already occurred. In our study, we observed a strong correlation between the predicted crRNA activity ranking of MPXV, a pathogen responsible for a recent outbreak of acute human viral disease [2], and the ranking derived from experimental results. This finding highlights the high utility of the deep learning model for crRNA design. Furthermore, our design and validation results for other pathogenic nucleic acid crRNAs, such as the viral targets of EV71 and CV‐A16, and the bacterial target of *L. monocytogenes*, further support the efficacy of this approach. In addition, our research demonstrates that EasyDesign (https://crispr.zhejianglab.com/) is sufficient to support the establishment of a routine clinical rapid detection system for different HPV subtypes (Figure 4C–H).

However, our current model training data is based on the use of the LbCas12a protein [7], which recognizes the PAM sequence “TTTN.” This means that the predictive performance of LbCas12a variants, as well as other members of the Cas12 protein family, requires further investigation and refinement. Future research will aim to extend these findings, enhancing our understanding of the broader applicability of these detection methods.

## CONCLUSION

In summary, we have developed a deep learning model called EasyDesign to facilitate rapid and highly efficient crRNA design for Cas12a‐based detection. In addition, we have created an online platform (https://crispr.zhejianglab.com/) for EasyDesign that integrates RPA primer design for ease of use. This has the potential to accelerate the development and application of Cas12a‐based technologies. In public health, EasyDesign, a rapid and easy‐to‐use approach, will help public health officials develop effective strategies to control and prevent the spread of disease.

## METHODS

### Design of DNA template and crRNA

We performed a global design of crRNA concerning the reported nucleic acid detection targets for different pathogens (Table S1) to maximize the generation of laboratory assay data. The DNA plasmids, which contained sequences adapted from viruses and bacteria, were synthesized by Sangon Biotech (Shanghai, China). After generating the crRNA sets, we differentiated the DNA templates to generate nine template sets for each original DNA template with mutation, insertion, and deletion. In detail, a total of 22 original sequence templates were used, and for each original sequence template, eight mutated sequence templates were designed, including the following: six mutated templates characterized by mutation of one base per 24, 27, and 20 nt lengths of the original template, counting from positions 20 or 22 nt from the 5′ end of the sequence; one deletion sequence template characterized by a deletion of one base every 40 nt in length, counting from a position 20 nt from the 5′ end of the sequence; one insertion sequence template characterized by a deletion of one base every 40 nt in length, counting from a position 20 nt from the 5′ end of the sequence. All the crRNAs were designed according to the principles of the LbCas12a crRNA characteristics. The crRNA candidates for the nucleic acid detection targets were designed with the PAM of “TTTN” and were synthesized by Genscript.

### Quantification of Cas12a cleavage activity

For activity quantification, the Cas12a‐based reaction mixture contained 200 ng purified LbCas12a, 25 pM ssDNA FQ probe sensor, 1 µM crRNA, and 2 μL the sample in a reaction buffer (100 mM NaCl, 50 mM Tris‐HCl, 10 mM MgCl_2_, 100 µg/mL bovine serum albumin, pH 7.5). The reaction volume was 20 μL, and the reaction was performed at 37°C. The cleavage results of the CRISPR reaction were analyzed using a Varioskan® LUX multimode microplate reader (Thermo Scientific). The excitation wavelength was set at 485 nm, and the fluorescence emission was collected at 535 nm. Negative and positive controls were included in the samples, and subsequent calculations included normalization of the fluorescence values using these controls. The microplate readers were configured to record fluorescence values every 2 min during the experiments. To meet the requirements of rapid detection, the fluorescence value at 30 min was selected as the indicator for activity assessment.

### Predicting detection activity: Data processing

In our experiment, we placed two wells of reaction components (no DNA template) as negative controls and two duplicate wells of an assay with high fluorescence activity as positive controls in each 96‐well plate. To eliminate the effect of background noise, we subtracted the mean value of the negative controls in the same plate from the plate reader readings and then divided it by the mean value of the positive control readings. This normalization process was applied to our model training data.

### Data augmentation

To minimize fluorescence value fluctuations in our experimental detection and normalize the data, we employed a time‐folding technique to introduce label value uncertainty into our deep learning model. For each target DNA and crRNA pair, two label values were used: (1) the fluorescence value recorded at 30 min and (2) the normalized fluorescence value at 30 min based on 20 min recorded data.

Assuming that the readings at *t*2 min are to be normalized to t1 min, the normalization method is

g(t1,t2)=[[f(t1+2) −f(t1−2)]^[(t1−t2)/4]]×f(t2).

Here, *f*(*x*) is the fluorescence readout at *x* minutes, and *g*(*t*1, *t*2) is the value of the fluorescence readout at the time of *t*2 normalized to the time of *t*1.

### One‐hot (2D) encoding

The one‐hot (2D) encoding for the base encoding. In the cases of insertions or deletions, a “‐” placeholder is used in the target DNA or crRNA sequence, resulting in five possible scenarios: “A,” “C,” “T,” “G,” and “‐.” The corresponding positions of the target DNA and crRNA together form a 10‐dimensional one‐hot vector. Thus, the input dimensionality is (45, 10) and consists of a concatenated one‐hot encoding of the target and guide sequences. Specifically, each element xi (*i* ϵ {1…45} *v*) is a vector [xi, *t*, xi, *g*]. The target context corresponds to *i* ϵ {1…10} (5′ end) and *i* ϵ {36…45} (3′ end); for these *i* values, xi, *t* is a one‐hot encoding of the target sequence, and xi, *g* is all 0. The guide binds to the target at *i* ϵ {11…35}, and for these *i* values, xi, *t* is a one‐hot encoding of the target sequence protospacer at position *i*‐10 where the guide is designed to bind, while xi, *g* is a one‐hot encoding of the guide at position *i*‐10. The CNN model uses the output of one‐hot vector, while in the transformer model, the vectors are then processed through a 1D Convolutional (Conv) layer, followed by the classic transformer encoder and decoder layers.

### Model selection

Previous studies on guide RNA performance prediction have investigated the performance of various deep learning models in addressing this issue, with data indicating that CNNs outperform LR, GBT, RF, SVM, LSTM, and traditional MLPs [10]. Therefore, our study primarily compares the performance of CNN models with Transformer models in addressing crRNA computational issues.

### CNN models: CNND, CNN12a, and CNN12ae

CNND is a convolutional neural network model derived from the Adapt framework [9]. It has been trained on our Cas12a data set, which is a diverse collection of samples representing various patterns. The network architecture consists of a convolutional block, a spreading layer (Flatten), and two dense layers (Dense). The convolutional block consists of a convolutional layer, a pooling layer, a batch normalization layer (BN layer), and a local connectivity layer. The convolutional layer in the regression model has a width of {1, 2}, with 25 convolutional kernels, a pooling layer width of 2, and the utilization of Average pooling. The fully connected layer has a dimension of 53, the locally connected layer has a width of {1, 2}, and a dimension of 3. The activation function used is ReLU, the dropout probability is set to 0.30, the batch size is 229, and no batch normalization is applied. In addition, no regression parameter pruning is performed.

CNN12a, an extension of CNND, incorporates two key modifications. First, the encoding mechanism has been adapted to better capture the underlying requirements of the Cas12a diagnostic scenarios. Specifically, the fluorescence value at 30 min indicates activity or inactivity, with the model tasked to identify the brightest fluorescing crRNA as the target. This adjustment results in a more robust feature representation capable of distinguishing between high‐quality and suboptimal crRNA. Secondly, the loss function used to calculate the discrepancy between predicted values and laboratory results has been revised. This updated loss function exhibits greater sensitivity to the subtleties within the data, providing better guidance to the model during training and consequently improving the prediction of diagnostic efficacy. CNN12a was trained on the same data set as CNND to ensure a fair comparison between the models.

CNN12ae, an enhanced version of CNN12a, focuses on data augmentation for addressing imbalanced data. Upon scrutinizing the training data, a significant imbalance in the sample distribution was observed, with a bias towards fully matching pairs. To solve this problem, data augmentation techniques were used to augment the data set with partially matching samples. Highly mismatched crRNA/target pairs labeled “inactive” were introduced synthetically by randomly introducing >4 mismatches into the existing data. This augmentation process also included data enhancement at the 20‐min time point. This resulted in a more balanced data set was obtained, which was subsequently used to train CNN12ae, an improved iteration of CNN12a.

### Transformer models: TransformerD, Transformer12a, and Transformer12ae

TransformerD was initially constructed based on previous research that had successfully used the transformer architecture to predict Cas13a targets [22]. We then fine‐tuned TransformerD using our data set and introduced essential modifications to create Transformer12a. These modifications included adjustments to the encoding method to account for mismatched samples. Additionally, we explored the incorporation of a convolutional layer before the encoding layer to extract intricate patterns and features from the input sequence. Furthermore, Transformer12a was trained to utilize the augmented data set, resulting in Transformer12ae.

### Data set preparation

In this study, we used a meticulously processed data set containing 11,496 pairs of experimental data related to crRNA and target DNA. This data set was used for both training and testing purposes. To more accurately replicate real‐world scenarios in which the model is tasked with providing crRNA recommendations for previously unseen target DNA (i.e., zero‐shot recommendations), we randomly partitioned the data set into two sets for training and testing. These sets were completely nonoverlapping. Specifically, the training set encompassed 20 distinct classes of DNA, comprising a total of 10,634 pairs, while the testing set consisted of 1358 pairs of data.

### Model selection and evaluation

Both Spearman's correlation (*ρ*) and Pearson's correlation (*r*) were used to assess the performance of the models. These metrics were used to measure the correlation coefficients between the model predictions and the outcomes obtained from actual experiments.

$$\rho =1-\frac{6\Sigma {\mathrm{d}}_{i}^{2}}{n\left(n^{2}-1\right)}.$$

Here, *d*_*i*_ **= difference between the two ranks of each observation; *n* = number of observations.

$$r=\frac{N\sum \mathrm{xy}-\left((,\sum x,)\right)\left((,\sum y,)\right)}{\sqrt{\left[N\sum x^{2}-{\left((,\sum x,)\right)}^{2}][N\Sigma y^{2}-{\left((,\sum y,)\right)}^{2}\right]}}.$$

Here, *N* = the number of pairs of scores; Σ*xy* = the sum of the products of paired scores; Σ*x* = the sum of *x* scores; and Σ*y* = the sum of *y* scores.

### Experimental validation of the developed model

DNA sequences commonly used for molecular detection of four pathogens were selected as input verses for experimental validation of the developed model, including MPXV, EV71, CV‐A16, and *L. monocytogenes*. The crRNA (Table S4) was designed globally based on the target DNA sequences of these four pathogens and then synthesized for CRISPR detection, and the fluorescence results were compared with the model‐predicted crRNA activity. We annotated the results with the qPCR detection sites of four pathogens to compare activity, including MPXV [25], EV71 [26], CV‐A16 [27], and *L. monocytogenes* [28]. The CRISPR assay steps are described in the section “Quantification of Cas12a cleavage activity.”

### RPA reaction

For the RPA reaction, GenDx Basic kits (KS101; GenDx Biotech) were used according to the manufacturer's instructions with slight modifications. Briefly, in a reaction tube, add 20 μL lysis reagent, 3 μL forward primer (10 μM), 3 μL reverse primer (10 μM), 5 μL template DNA (varies depending on the application), and 17 μL ddH_2_O for a total volume of 48 μL, shake well and centrifuge instantly. For each sample, add 2 μL activator (magnesium acetate) to the tube, close the cap carefully, shake well, and centrifuge immediately. Place the tubes in a thermostat and incubate at 39°C for 25 min. The RPA product obtained was used for downstream applications.

### Principles of RPA primer design

The RPA primers for the nucleic acid detection targets were designed following the instructions from TwistDx (http://www.twistdx.co.uk). The main design principles include the following: The GC content of the primers is between 35% and 60%. No more than four consecutive base forms of A, T, C, and G within the primer. No G within the 3 nt interval at the 5′ terminal of the primer. The primers contain G or C within the 3 nt interval at the 3′ terminal.

### Clinical samples and the qPCR identification

The human cervical cell specimens (*n* = 15) used in this study were collected from the First Affiliated Hospital, Zhejiang University School of Medicine. These positive samples were screened for HPV subtypes by qPCR method (Liferiver) in the clinical laboratory [29]. The samples were anonymized and did not contain any personal identification data of individuals. The cervical cell precipitates were resuspended after centrifugation using lysate buffer for nucleic acid preparation. The samples were then heated at 95°C for 10 min to release HPV DNA and stored at −20°C before use.

### CRISPR detection of clinical sample

For the CRISPR reaction, we prepared a 20 µL CRISPR reaction system by mixing the components as follows: 8.8 μL ddH_2_O, 2 μL reaction buffer, 0.2 μL RNAase inhibitor (Novoprotein), 1.0 μL ssDNA FQ reporter (Azenta), 2 μL subtype‐specific crRNA (Genscript), 1.0 μL LbCpf1 protein. Then, 5 µL DNA template was added to the CRISPR reaction. Then, the 96‐well plate was loaded into the carrier of the Varioskan™ LUX multimode microplate reader (Thermo Scientific), and the fluorescence values were collected every 2 min for 2 h at 37°C. Simultaneously, the fluorescence signal of the reaction in the tube was photographed after 30 min.

## AUTHOR CONTRIBUTIONS

Xinjie Wang, Xingxu Huang, Baicheng Huang, and Ling Guo conceived and designed the project. Baicheng Huang, Ling Guo, Hang Yin, Yue Wu, and Zihan Zeng performed most experiments with the help of Zhimin Ai, Weiqiang Zhang, Xingchi Kan, Sujie Xu, Qian Yu, Shimin Du, and Lina Wu. Yufeng Lou provided expert technical assistance. Baicheng Huang and Ling Guo wrote the paper with helps from all authors. Xinjie Wang, Chao Li, Shengqi Wang, and Xingxu Huang revised the manuscript and managed the project. All authors have read the final manuscript and approved it for publication.

## CONFLICT OF INTEREST STATEMENT

The authors declare no conflict of interest.

## ETHICS STATEMENT

The detection of HPV clinical samples was approved by the Clinical Research Ethics Committee of the First Affiliated Hospital, Zhejiang University School of Medicine (No. IIT2023014A). All research was performed following relevant guidelines and regulations. Informed consent was obtained from all participants.

## Supporting information


**Figure S1:** Distribution of base types at different positions in the crRNAs set.
**Figure S2:** Schematic flowchart of the CNN and Transformer models used in the training.
**Figure S3:** The point density map and ridgeline plot of predicting activities of guide–target pairs by the models of CNN and transformer.
**Figure S4:** Fluorescence kinetic curves for high mismatch guide–target pairs.
**Figure S5:** Correlation analysis of characteristics and activity of guide‐to‐target pairs.
**Figure S6:** Predictive performance of CNN12ae in viral and bacterial pairs.
**Figure S7:** CRISPR fluorescence results at different DNA template concentrations.
**Figure S8:** The fluorescence kinetic curve of the Cas12a reaction in the detection of HPV clinical samples.

**Table S1:** Information on the types of viral and bacterial species used to build crRNA libraries.
**Table S2:** Information on viral and bacterial nucleic acid templates used to build crRNA libraries.
**Table S3:** Training data datasets for model development generated based on CRISPR fluorescence reaction.
**Table S4:** Augment datasets for model development generated based on CRISPR fluorescence reaction.
**Table S5:** Test datasets for model development generated based on CRISPR fluorescence reaction.
**Table S6:** Model performance evaluation by k‐fold cross‐validation.
**Table S7:** The DNA templates of four pathogens (MPXV, EV71, CV‐A16, and *L. monocytogenes*) were used in the experimental activity testing.
**Table S8:** The crRNAs of four pathogens (MPXV, EV71, CV‐A16, and *L. monocytogenes*) were used in the experimental activity testing.
**Table S9:** Optimal HPV crRNAs and RPA primers generated from the one‐stop web platform of EasyDesign.

## Data Availability

Code is available in several repositories: EasyDesign is freely available at https://github.com/scRNA-Compt/EasyDesign; Scripts to install and replicate the model are available at https://github.com/scRNA-Compt/EasyDesign/blob/main/README.md; An online website providing instant Cas12a design is available at: https://crispr.zhejianglab.com/. Supplementary materials (figures, tables, scripts, graphical abstract, slides, videos, Chinese translated version, and updated materials) may be found in the online DOI or iMeta Science http://www.imeta.science/.
