# Supplementary material for: Deep learning enhancing guide RNA design for CRISPR/Cas12a‐based diagnostics
Source: Imeta. 2024 Jun 15;3(4):e214. doi: 10.1002/imt2.214 (PMC11316927; doi:10.1002/imt2.214)
Supplement: Supplementary file 1 — Figure S1: Distribution of base types at different positions in the crRNAs set. Figure S2: Schematic flowchart of the CNN and Transformer models used in the training. Figure S3: The point density map and ridgeline plot of predicting activities of guide–target pairs by the models of CNN and transformer. Figure S4: Fluorescence kinetic curves for high mismatch guide–target pairs. Figure S5: Correlation analysis of characteristics and activity of guide‐to‐target pairs. Figure S6: Predictive performance of CNN12ae in viral and bacterial pairs. Figure S7: CRISPR fluorescence results at different DNA template concentrations. Figure S8: The fluorescence kinetic curve of the Cas12a reaction in the detection of HPV clinical samples. [file IMT2-3-e214-s002.pdf]

Supporting information to

Deep learning enhancing guide RNA design for CRISPR/Cas12a-based diagnostics

Running title: Smart guide RNA design for CRISPR/Cas12a diagnostics

Baicheng Huang[1#], Ling Guo[1#], Hang Yin[1], Yue Wu[1], Zihan Zeng[1], Sujie Xu[1], Yufeng Lou[2], Zhimin Ai[1], Weiqiang Zhang[1], Xingchi Kan[1], Qian Yu[1], Shimin Du[1], Chao Li[3], Lina Wu[4], Xingxu Huang[1], Shengqi Wang[5*], Xinjie Wang[6*]

[1] Zhejiang Laboratory, Hangzhou, 311121, China

[2] Department of Laboratory Medicine, the First Affiliated Hospital, Zhejiang University School of Medicine; Key Laboratory of Clinical In Vitro Diagnostic Techniques of Zhejiang Province; Institute of Laboratory Medicine, Zhejiang University, Hangzhou, 311121, China

[3] Department of Applied Mathematics and Theoretical Physics, University of Cambridge, Wilberforce Road, Cambridge, CB3 0WA, UK.; School of Medicine, School of Science and Engineering, University of Dundee, Nethergate, Dundee, DD1 4HN, UK

[4] School of Food Science and Pharmaceutical Engineering, Nanjing Normal University, Nanjing, 210023, China

[5] Bioinformatics Center of AMMS, Beijing 100850, China

[6] Shenzhen Branch, Guangdong Laboratory of Lingnan Modern Agriculture, Genome Analysis Laboratory of the Ministry of Agriculture and Rural Affairs, Agricultural Genomics Institute at Shenzhen, Chinese Academy of Agricultural Sciences, Shenzhen, 518000, China

[#]These authors contributed equally: Baicheng Huang, Ling Guo
*Correspondence: wangxinjie@caas.cn (Xinjie Wang); sqwang@bmi.ac.cn (Shengqi Wang)

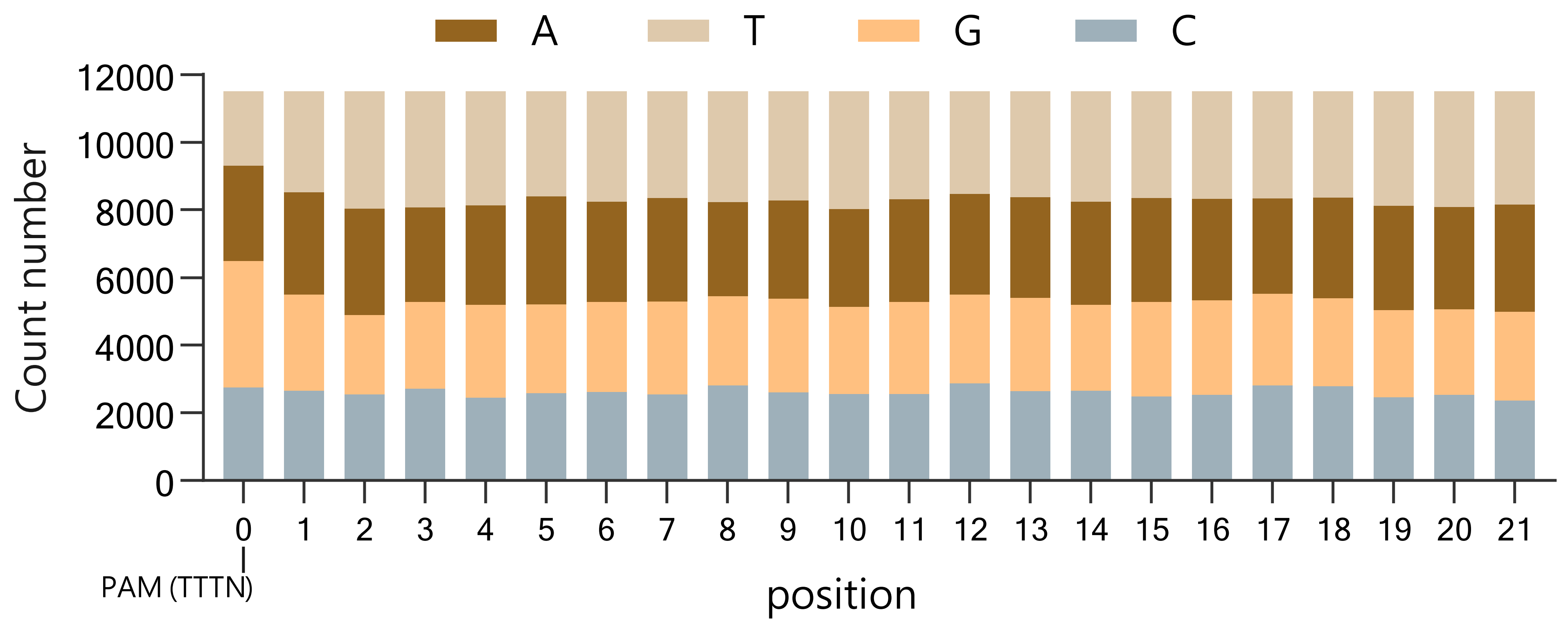

Figure S1. Distribution of base types at different positions in the crRNAs set.

Distribution of the number of base types at each position of crRNA, with different colors indicating A, T, C, and G, for a total of 11,496 guide-to-target pairs.

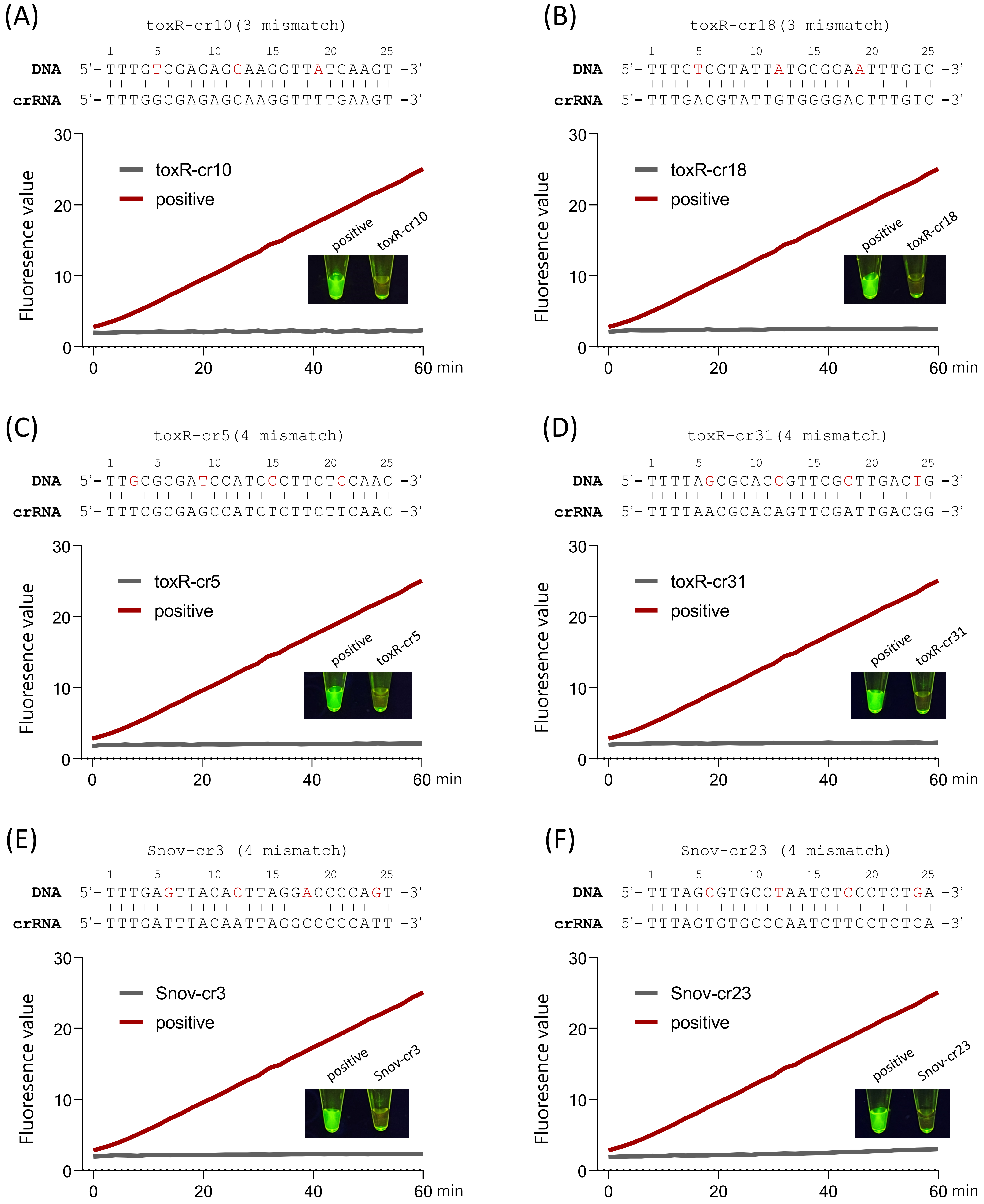

Figure S2. Fluorescence kinetic curves for high mismatch guide–target pairs.

Kinetic curves of CRISPR-fluorescence data of 6 high mismatch guide-to-target pairs, including the case of 3 (**A-B**) and 4 (**C-F**) mismatches in the guide-to-target pairs.

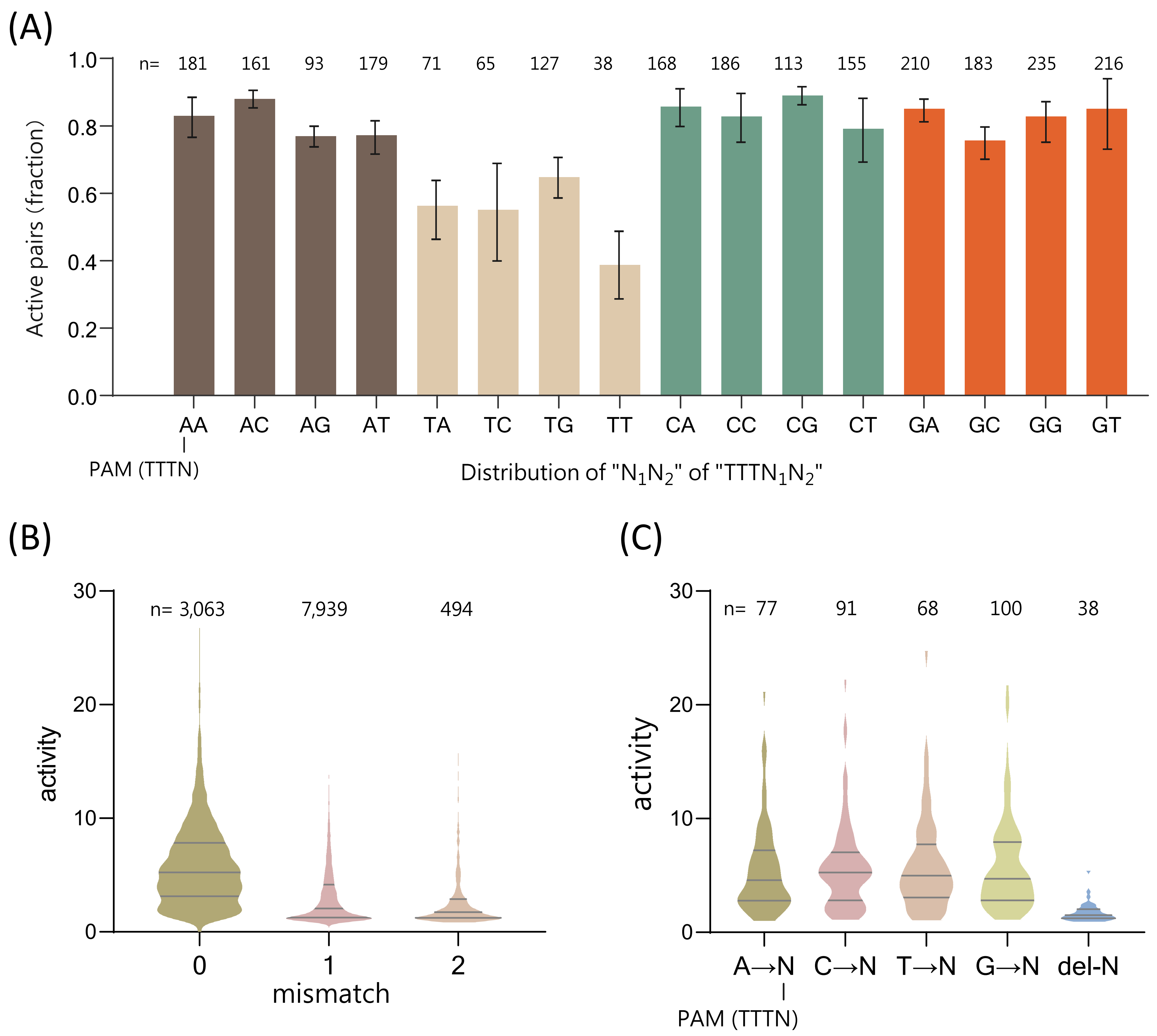

Figure S3. Correlation analysis of characteristics and activity of guide-to-target pairs.

(A) Fraction of active guide-to-target pairs for each 2-nt flanking site of "TTTN1-N2" of the PAM and an extending position. (B) Guide-to-target pairs activity statistics for different mismatch scenarios. (C) Guide-to-target pairs activity statistics for different mutation scenarios of "N" in PAM "TTTN".

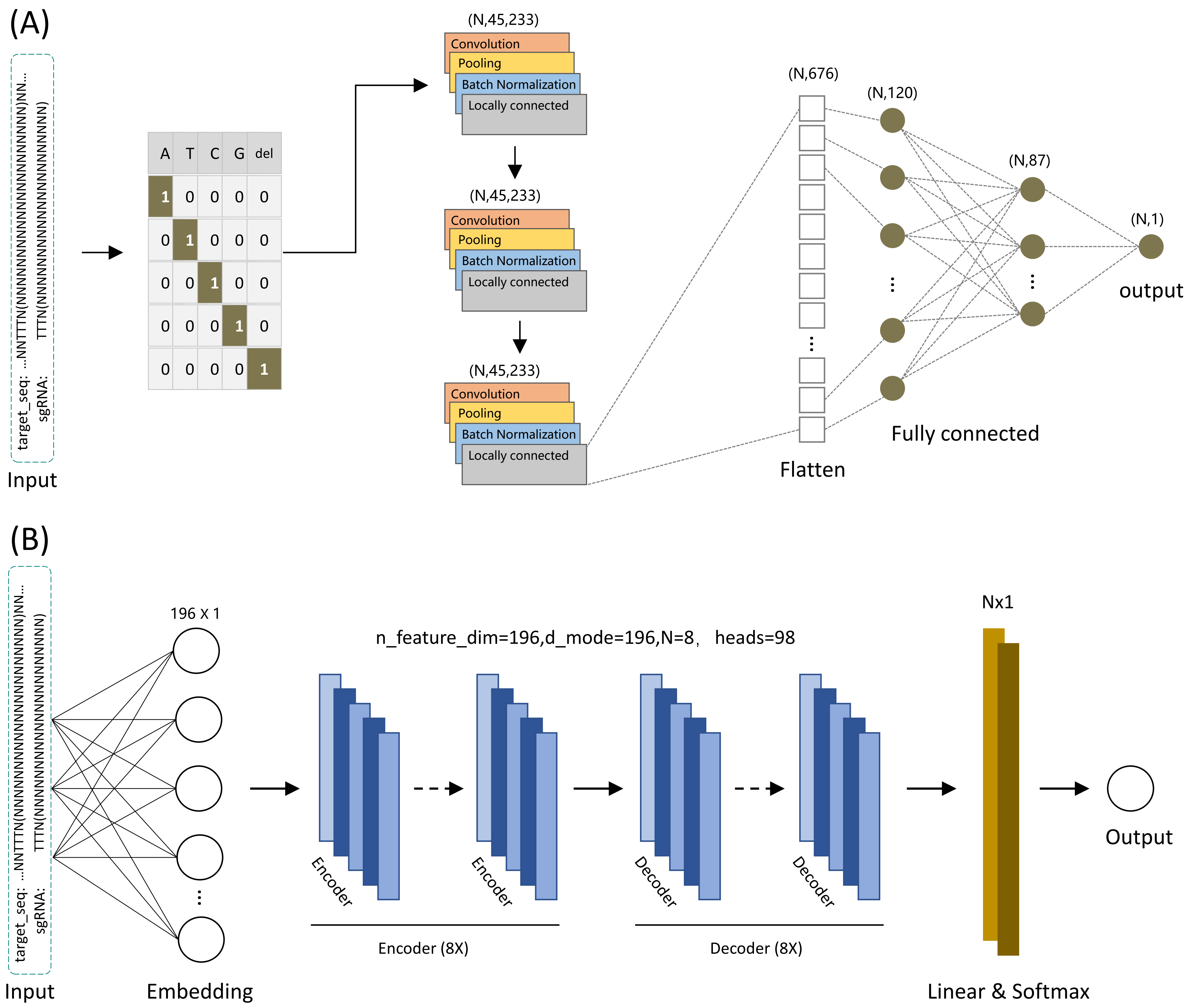

Figure S4. Schematic flowchart of the CNN and Transformer models used in the training.

(A) In CNN we have a total of N pairs of sgRNA/target sequences, which are first one-hot encoded. The input data is initially passed through a convolutional block. The convolutional blocks results in output data (N, 45, 233), where N represent the count of input pairs, and 45 and 233 represent the dimensions of the output. The feature maps are flattened, converting into a one-dimensional vector (N, 676), which is passed through several fully connected layers, and resulting in a (N,1) vector with 1 prediction value for each pair. (B) The architecture of the transformer model consists of an embedding layer, 8 encoder stacks, and 8 decoder stacks. Initially, each sequence pair is transformed into a 196x1 vector by the embedding layer. This vector representation serves as the input to the encoding layers. Both the encoder and decoder contain 8 layers, and the model has a dimension of 196 (n_feature_dim=196 and d_model=196). A multi-head self-attention mechanism with 98 attention heads is employed within each layer. The output from the last decoder layer is passed through a linear softmax layer, which reduces the dimensionality from 196 dimensions to an Nx1 vector. This Nx1 vector represents the model's predictions for each input sequence pair.

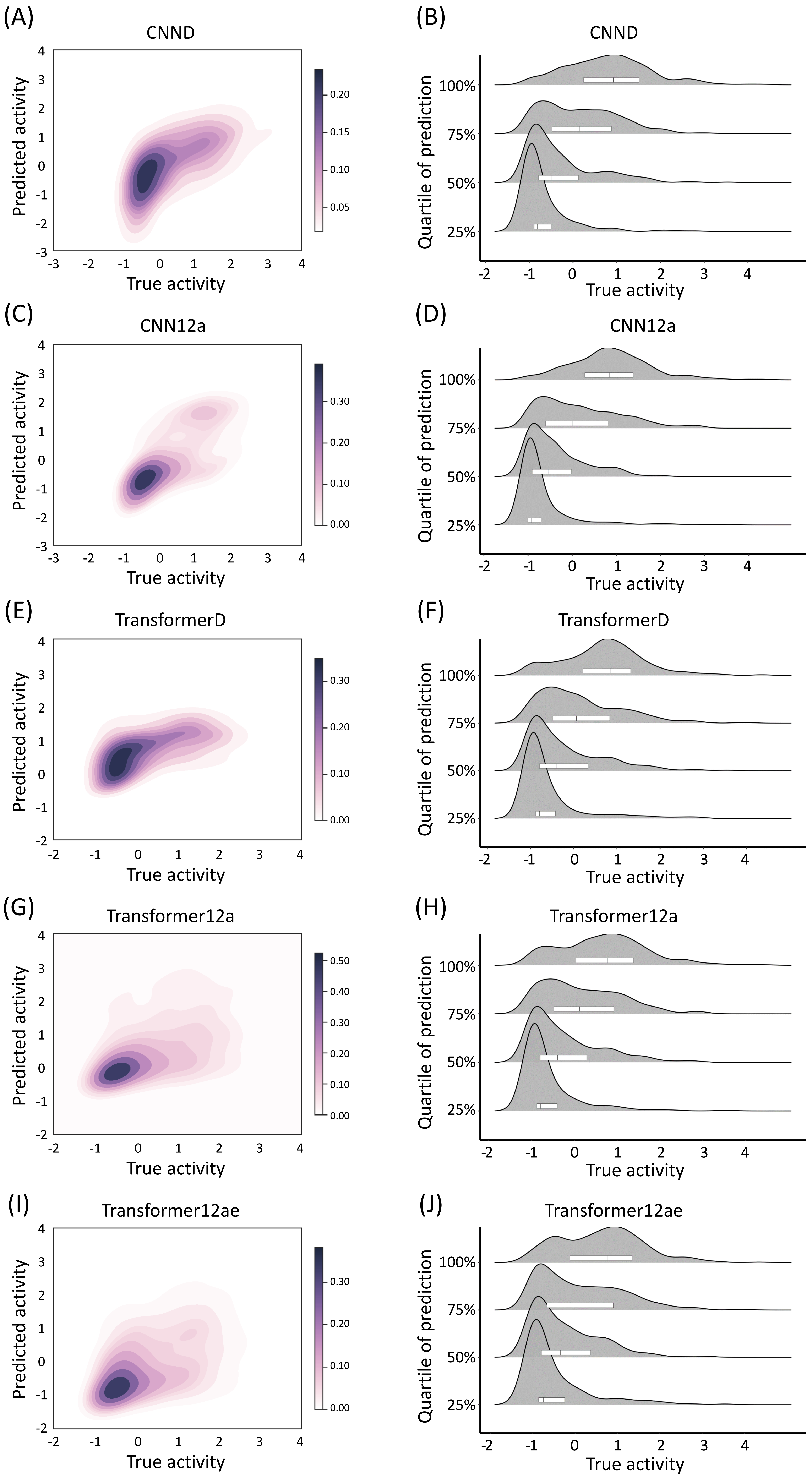

Figure S5. The point density map and ridgeline plot of predicting activities of guide–target pairs by the models of CNN and Transformer. Point density maps and ridgeline plot for predicting the activity of guide - target pairs by CNN and transformer models, including 2 models derived from CNN (CNND, **A-B**; CNN12a, **C-D**) other than CNN12ae, and 3 models derived from Transformer (TransformerD, **E-F**; Transformer12a, **G-H**; Transformer12ae, **I-J**).

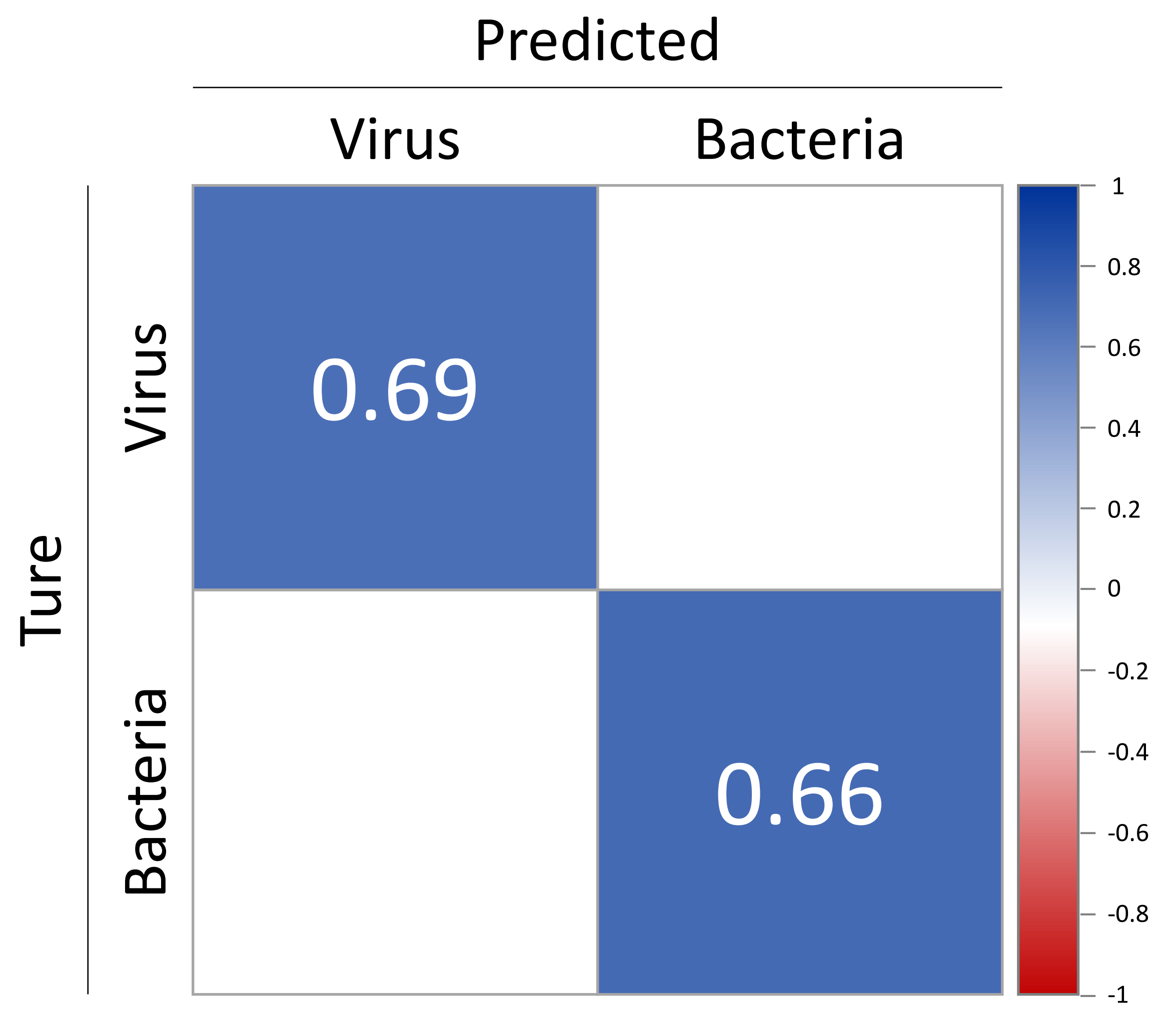

Figure S6. Prediction performance of CNN12ae in viral and bacterial guide-to-target pairs.

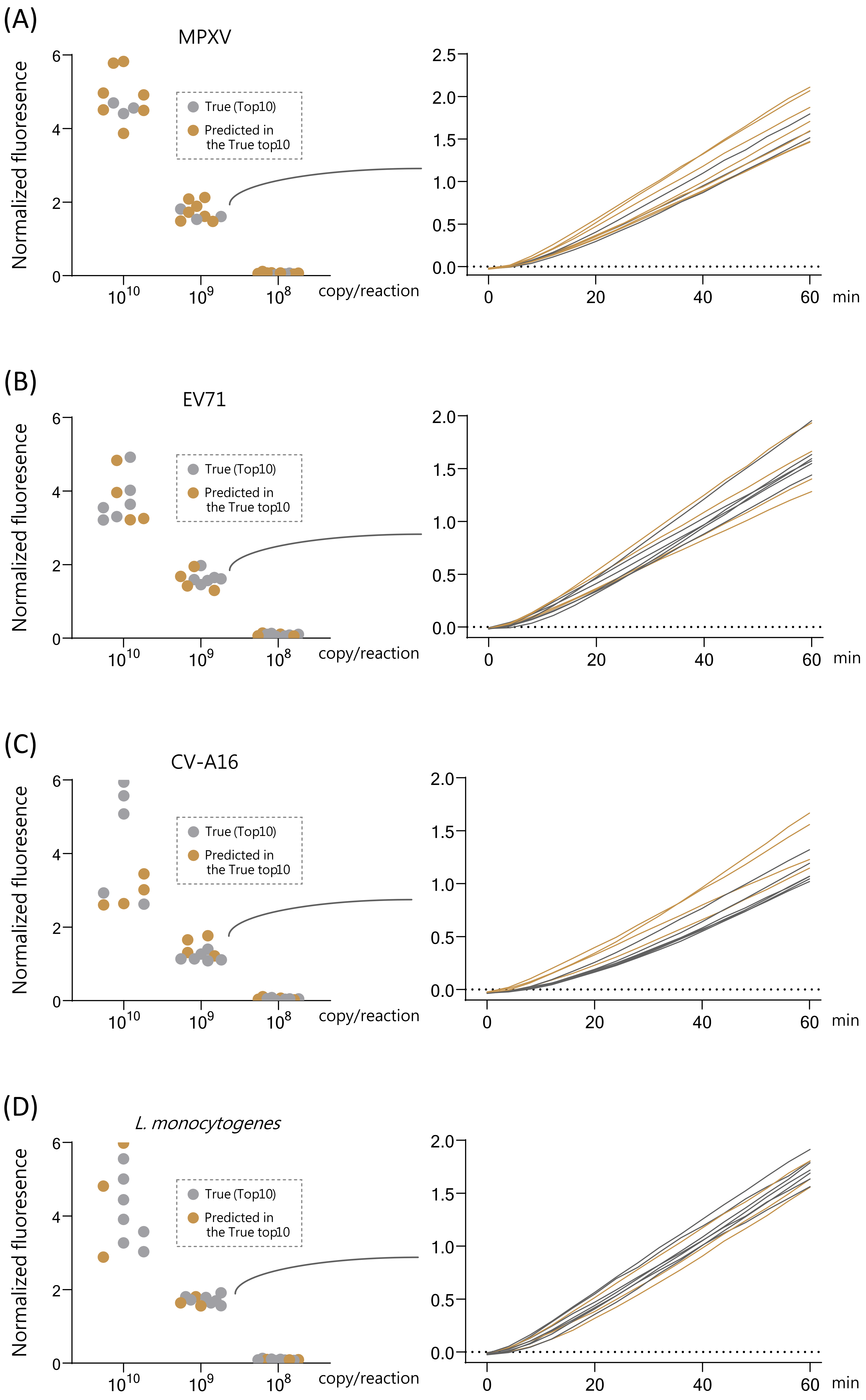

Figure S7. CRISPR fluorescence results at different DNA template concentrations. The distribution of CRISPR fluorescence results for the four DNA templates (MPXV, **A**; EV71, **B**; CV-A16, **C**; *L. monocytogenes*., **D**) used to validate the optimal deep learning model CNN1e at different concentrations, including 1 × 10^{10}, 1 × 10^{9} and 1 × 10^{8}.

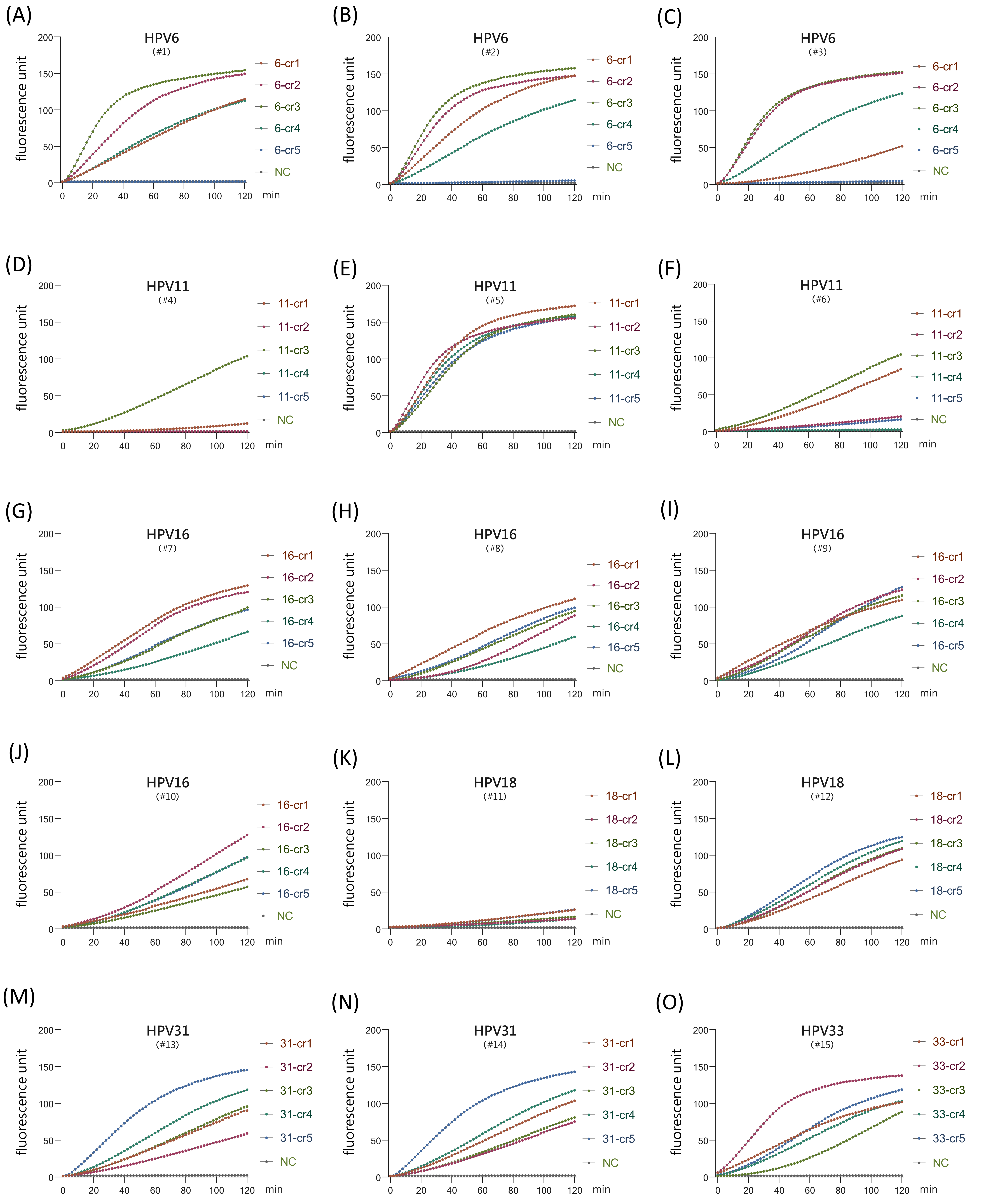

Figure S8. The fluorescence kinetic curve of the Cas12a reaction in the detection of HPV clinical samples. Fluorescence kinetic curves for the Cas12a-based CRISPR reaction in the detection of HPV clinical samples. Positive clinical samples (15 in total, **A-O**) of six HPV subtypes were tested, including HPV6 (3 samples), HPV11 (3 samples), HPV16 (4 samples), HPV18 (2 samples), HPV31 (2 samples) and HPV33 (one sample). Each sample was tested using the five preferred crRNAs predicted by the one-stop web platform and kinetic curves were generated separately.
